# Proton vs photon: A model-based approach to patient selection for reduction of cardiac toxicity in locally advanced lung cancer

**DOI:** 10.1016/j.radonc.2019.06.032

**Published:** 2020-11

**Authors:** S. Teoh, F. Fiorini, B. George, K.A. Vallis, F. Van den Heuvel

**Affiliations:** a CRUK/MRC Oxford Institute for Radiation Oncology, Old Road Campus Research Building, University of Oxford, Oxford, OX3 7DQ, UK; b Department of Radiotherapy, Oxford Cancer Centre, Oxford University Hospitals NHS Foundation Trust, OX3 7LE, UK

**Keywords:** Cardiac toxicity, Proton therapy, Lung cancer, VMAT

## Abstract

**Purpose/objective:**

To use a model-based approach to identify a sub-group of patients with locally advanced lung cancer who would benefit from proton therapy compared to photon therapy for reduction of cardiac toxicity.

**Material/methods:**

Volumetric modulated arc photon therapy (VMAT) and robust-optimised intensity modulated proton therapy (IMPT) plans were generated for twenty patients with locally advanced lung cancer to give a dose of 70 Gy (relative biological effectiveness (RBE)) in 35 fractions. Cases were selected to represent a range of anatomical locations of disease. Contouring, treatment planning and organs-at-risk constraints followed RTOG-1308 protocol. Whole heart and ub-structure doses were compared. Risk estimates of grade⩾3 cardiac toxicity were calculated based on normal tissue complication probability (NTCP) models which incorporated dose metrics and patients baseline risk-factors (pre-existing heart disease (HD)).

**Results:**

There was no statistically significant difference in target coverage between VMAT and IMPT. IMPT delivered lower doses to the heart and cardiac substructures (mean, heart V5 and V30, P < .05). In VMAT plans, there were statistically significant positive correlations between heart dose and the thoracic vertebral level that corresponded to the most inferior limit of the disease. The median level at which the superior aspect of the heart contour began was the T7 vertebrae. There was a statistically significant difference in dose (mean, V5 and V30) to the heart and all substructures (except mean dose to left coronary artery and V30 to sino-atrial node) when disease overlapped with or was inferior to the T7 vertebrae. In the presence of pre-existing HD and disease overlapping with or inferior to the T7 vertebrae, the mean estimated relative risk reduction of grade⩾3 toxicities was 24–59%.

**Conclusion:**

IMPT is expected to reduce cardiac toxicity compared to VMAT by reducing dose to the heart and substructures. Patients with both pre-existing heart disease and tumour and nodal spread overlapping with or inferior to the T7 vertebrae are likely to benefit most from proton over photon therapy.

## Highlights

• Dose to the heart and all its substructures were significantly lower with IMPT compared to VMAT.
• Our analysis showed that not all patients benefit equally from proton beam therapy.
• Patients with disease involvement overlapping with or are inferior to the most superior aspect of the heart contour appear to have the highest benefit. The median level of the superior aspect of the heart contour began was T7 vertebra.

Outcome of patients with locally advanced non-small cell lung cancer (NSCLC) (stage III) is poor. In RTOG 0617, a median survival of less than 28 months is reported following radical chemoradiotherapy [1]. Efforts to improve survival through dose escalation have been unsuccessful and in fact appeared to be detrimental. Increased cardiac dose was implicated as one of the reasons for this. Since RTOG-0617 was reported, growing evidence shows that cardiac morbidity and mortality secondary to radiotherapy occurs much earlier than previously thought [2], [3], [4], [5], [6]. In a multivariable analysis of RTOG-0617, higher radiation dose to the heart was independently associated with worse survival [1]. Dess et al., retrospectively evaluated the association between cardiac events and heart dose in four prospective RT trials in NSCLC. Pre-exisiting heart disease (HD) and higher heart dose were significantly associated with grade⩾3 cardiac events, with 10 and 15% risk of grade⩾3 cardiac events reported with mean heart dose (MHD) of 5 and 12 Gy respectively [3]. Wang et al., showed that heart doses, coronary artery disease and a higher baseline risk for heart disease were associated with cardiac events [6]. In their cohort, there was 21% risk of cardiac complication when MHD exceeded 20 Gy. The exact mechanism for radiation-induced heart disease (RIHD) in lung cancer is unknown but likely to be multifactorial. Clinical manifestations include coronary artery disease, pericardial disease and arrhythmia [7].

Proton beam therapy (PBT) could potentially improve outcome in these patients by reducing RIHD compared to photon therapy. However, patient selection is key to exploiting this technology. PBT is unlikely to improve outcome in cases where doses to the normal tissue and target are similar for both treatment modalities. Furthermore, even when dosimetric advantages are observed [8], [9], [10], [11], these do not necessarily translate into clinically meaningful benefit [12]. Patient-, disease- and treatment-related factors play a role in determining the outcome.

Model-based patient selection is one approach to defining which sub-group of patients would receive the largest gain from PBT [13]. Nevertheless, choosing the appropriate model is crucial. Most NTCP models rely only on dose parameters to estimate complication probabilities [14] and this limits their predictive power [15]. Incorporating risk factors into these models has been shown to improve their performance [15], [16].

We hypothesise that PBT could reduce dose to the heart and its substructures and therefore reduce cardiac complications without compromising tumour control in patients with locally advanced lung cancer. The study aim was to identify a sub-group of patients who would benefit from intensity modulated proton therapy (IMPT) over photon volumetric modulated arc therapy (VMAT) with respect to cardiac sparing. Identification of this sub-group would ultimately be useful in informing future clinical trial design of proton vs photon therapy in locally advanced lung cancer.

## Materials and methods

### Patients

Twenty NSCLC proxy patients were selected to provide a range of anatomical locations of primary tumours and nodal involvement (10/20 patients had left sided primary tumour, 11 had middle/lower lobe primary tumours). Most cases had nodal/mediastinal involvement as the main cohort of patients receiving radical chemoradiotherapy are stage III NSCLC (16/20). Of twenty cases, fourteen were previously treated with photon radiotherapy at our institution. The use of patient data was approved by the NHS Health Research Agency and conducted under the auspices of Oxford University Clinical Trials and Research Governance (research ethics committee reference: 16/LO/1324). The data for six more patients were provided by Hugo et al. [17] through the cancer imaging archive (TCIA) [18].

### Target structures and OAR

For each case, a dual-arc VMAT and mini-max robust-optimised (MM)-IMPT plans was created to a prescribed dose of 70 Gy (relative biological effectiveness (RBE)) in 35 fractions. Proton RBE was assumed to be 1.1. Four-dimensional (4D) CT simulation datasets were acquired for all plans. For treatment planning, an unweighted averaged-intensity projection (Ave-CT) dataset was generated. Target and organs-at-risk (OAR) delineation, and dose constraints were based on RTOG-1308 [19], [20]. The internal target volume (ITV) method was used to account for motion. Using this method, the gross tumour volume (GTV) was contoured in all 4D-CT phases and all the GTVs were combined to form the ITV. An 8 mm expansion of the ITV formed the clinical target volume (CTV). CTV was edited so that it did not cross anatomical boundaries unless there was tumour invasion. The planning target volume (PTV) was generated for VMAT plans following a 5 mm symmetrical expansion of CTV. Further details of the derivation of this margin can be found in the Appendix under treatment planning section.

The heart and the following substructures were delineated according to RTOG-1106 [21]: right and left: atria (RA,LA), ventricles (RV, LV) and coronary arteries (RCA, LCA), and sino-atrial node (SA node). An additional 3 mm margin was added to the coronary arteries to account for contouring variability. The SA node, which is found in the RA at the border of superior vena cava (SVC) opening, was defined as the superior 0.5 cm part of the right atrium plus an additional 0.5 cm of the inferior part of the SVC.

### Treatment planning

Different approaches were employed for VMAT and IMPT plans as IMPT plans are sensitive not only to setup but also range uncertainties which needed to be accounted for during the treatment planning stage in order to ensure adequate target coverage. VMAT plans were created with 6MV photons normalised to cover 95% of the PTV with the prescription dose. As no PTV was formed for IMPT, plans were normalised to cover 99% of the CTV with the prescription dose. The beam model used was based on an IBA facility at Provision Proton Therapy Centre, Knoxville, TN [22]. IMPT plans used multi-field optimisation with three to four beams (beam arrangements and use of range shifter can be found in Appendix Table A1). The robust optimisation parameters for setup and range uncertainties were 3 mm and 3.5% respectively. IMPT plans were optimised to the CTV.

In both treatment modalities, when constraints were met, plans were optimised to reduce dose to the OAR to as low as achievable while maintaining target coverage. Plans were created in Raystation treatment planning system v6.99 (Raysearch Laboratories, Stockholm). Optimisation of proton plans was done using Monte Carlo dose engine (v4.1) using 1% statistical uncertainty and a sampling history of 10,000 ions/spot. We assumed an end-to-end tumour motion of less than 10 mm in all cases, therefore an ITV approach based on the union of all the GTVs of all phases was used for planning for both VMAT and IMPT plans. For IMPT plans, strategies to mitigate the interplay effect, such as rescanning, would need to be implemented to ensure target coverage [23].

### Estimation of clinical benefit

The following dosimetric parameters were compared between VMAT and IMPT: MHD, volume of heart receiving 50 Gy(RBE), 30 Gy(RBE) and 5 Gy(RBE) (V50, V30 and V5), mean dose to the atria, ventricles, coronary arteries and SA node.

Grade⩾3 cardiac toxicities were estimated using a model which considered patients’ baseline cardiac morbidities and heart dose parameters [3]. Grading of cardiac complications was done retrospectively in the context of prospective trials using Common Terminology Criteria for Adverse Events (v4). The cardiac events recorded were: acute coronary syndrome, cardiac arrest, congestive heart failure (CHF), pericardial effusion, pericarditis, valvular disease and arrhythmia. The authors developed a Fine and Gray [24] competing risk regression models for predicting grade⩾3 cardiac toxicities at 24 months based on 125 patients enrolled in four prospective trials within a single centre. When non-cardiac death was accounted for as a competing risk, the 12- and 24-month cumulative incidence of ⩾grade 3 cardiac events were 9% (95%CI, 3–12%) and 11% (5–16%) respectively.

Pre-existing HD was associated with a higher cumulative incidence of cardiac events. The cumulative incidence without vs with pre-existing HD at 12 months was 15% (95% CI; 3–27%) vs 21% (7–35%) and at 24 months was 4% (0–9%) vs 7% (1–13%). Nomograms were available for predicting complications based on heart dose metrics (mean, V30 and V5) and the presence of pre-existing HD. Pre-existing HD was defined as a history of acute myocardial infarction, coronary artery bypass grafting procedure, angioplasty or stent placement, diagnosis coronary artery disease (CAD) or clinical diagnosis of CHF. In patients without known pre-existing HD, the likelihood of grade⩾3 events was further stratified based on patients baseline cardiac risk using the Framingham risk scores [25].

We estimated the predicted grade⩾3 toxicities for both treatment modalities in three different scenarios: in the presence of pre-existing HD, high risk of HD, and in the absence of pre-existing HD.

### Statistical analysis

Conformity indices (95% isodose volume/ CTV volume) were calculated for both treatment modalities. Spearman’s rank correlation co-efficient was calculated between heart dose and the thoracic vertebral level to which the most inferior aspect of the disease extended (primary tumour and nodes). Wilcoxon sign-rank test was used to compared the conformity indices, dose metrics and the absolute risk reduction between the treatment modalities. Statistical significance was defined as P < 0.05. All statistics were performed in IBM SPSS Statistics v20 (IBM Corp, Armonk, NY).

### Sample size and power calculation

A power calculation was performed based on the randomized controlled trial between intensity modulated radiotherapy (IMRT) and passive scatter proton therapy (PSPT) in lung cancer [12]. The median MHD of patients treated in the latter part of the trial for IMRT and PSPT were 10.4 Gy (range 0.9–34.6) and 5.5 Gy(RBE) (0.5–17) repsectively. The minimum sample size required to achieve power of 95% and a significance level of 5% for detecting a mean of the differences of 4.9 Gy(RBE) between the pairs was 13. Based on this trial, we defined a threshold of a difference of at least 5 Gy(RBE) to be clinically meaningful.

## Results

### Disease characteristics and target coverage

**Fig. A1:**
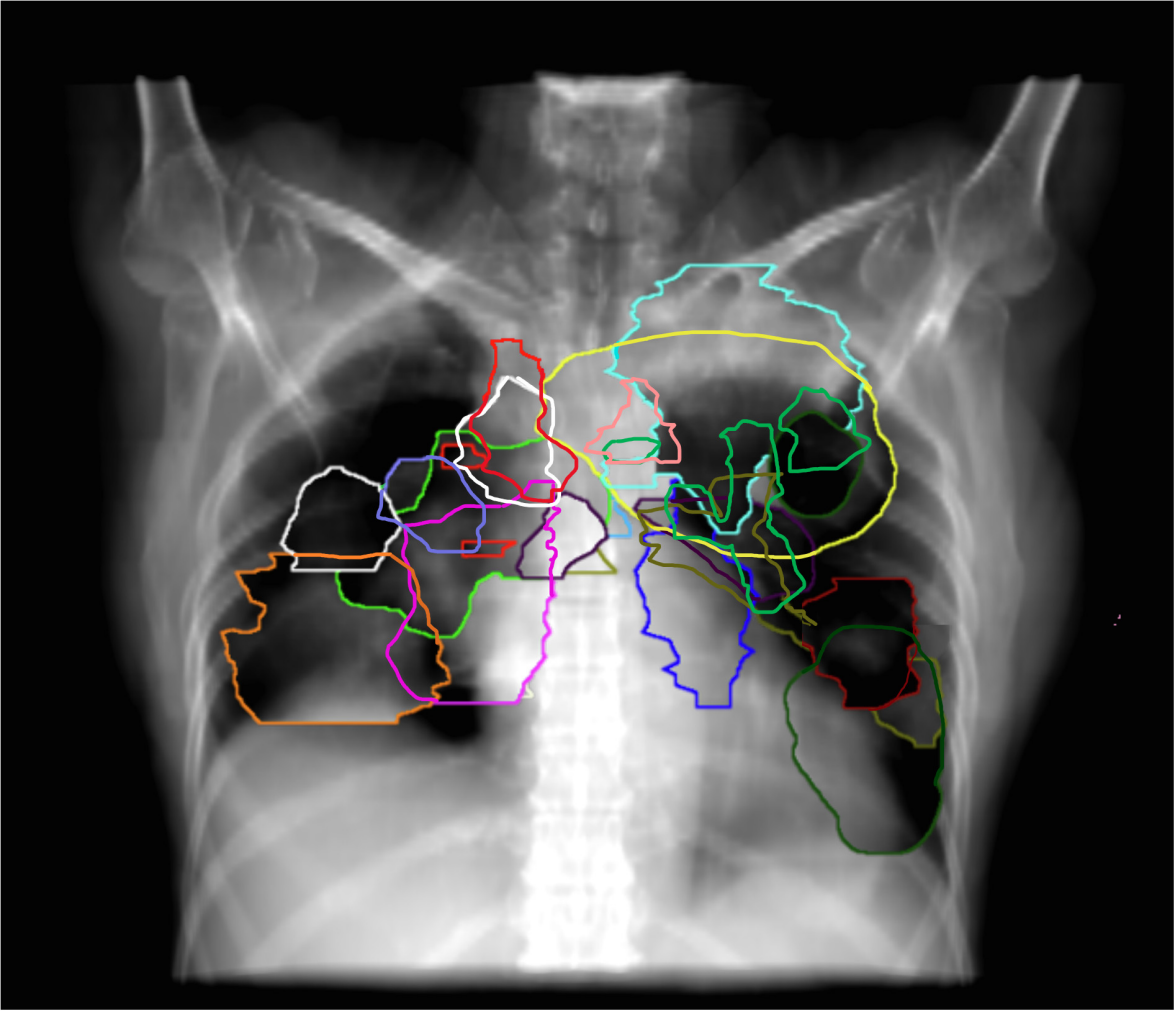
Coronal view of disease location of cases included in study.

The anatomical distribution of the primary tumour and the lymph node stations along with the TNM 8 staging included in this study can be found in Table 1 (see Appendix Fig. A1 for coronal view of disease locations). Tumour volume ranged from 15–404 cc. The majority of patients were stage III (16/20). Out of 16, 4 had T4N0 disease. These patients do not have nodal involvement but two had large tumours with mediastinal invasion (patient 5 – GTV 404 cc, patient 19 – GTV 306 cc), one had pericardial invasion (patient 7) and one was classified as stage III due to the presence of two tumours in the ipsilateral lung (patient 18). There was no statistically significant difference in target coverage between VMAT and IMPT. There was no statistically significant difference in conformity indices between VMAT and IMPT plans (VMAT vs IMPT, median (range): 1.92 (1.47–2.64) vs 2.03 (1.33–2.80), P = .351).

**Table 1:** Details of the twenty proxy cases. (GTV – gross tumour volume (includes primary and nodal spread), IASLC – International Association for the Study of Lung Cancer, LUL – left upper lobe, LLL – left lower lobe, RUL – right upper lobe, RML – right middle lobe, RLL – right lower lobe, * -* 2 separate primary tumour nodules found in left lung.

Patient	GTV (cc)	TNM 8	Primary tumour	IASLC Lymph node	Disease extension
		staging	locations	stations	(thoracic vertebrae level)
1	15	TxN2	–	5	6
2	261	T4N2	LUL	7, 10L	5
3	106	T2N0	RML	–	10
4	25	TxN2	–	10R, 4R	6
5	404	T4N0	LUL	–	8
6	50	T2N2	RUL	4R	6
7	21	T4N0	RUL	–	7
8	28	T1N2	LUL	10L, 4L	7
9	127	T2N2	RUL	10R, 7	9
10	56	T3N0	LLL	–	11
11	46	T3N2	LLL	7, 10L	9
12	50	T3N2	RLL	4R	8
13	48	T2N3	LUL	7, 10R	8
14	32	T3N0	RLL	–	10
15	115	T3/4 N1	RLL	10R	8
16	33	T2N1	LLL	10L	9
17	175	T3N2	RLL	7, 8	11
18	27	T2N0*	LLL	–	10
		T1N0*			
19	306	T4N0	RLL	–	10
20	68	T4N3	LLL	4L, 4R, 2Rx2	9

### Heart dose

**Fig. 1:**
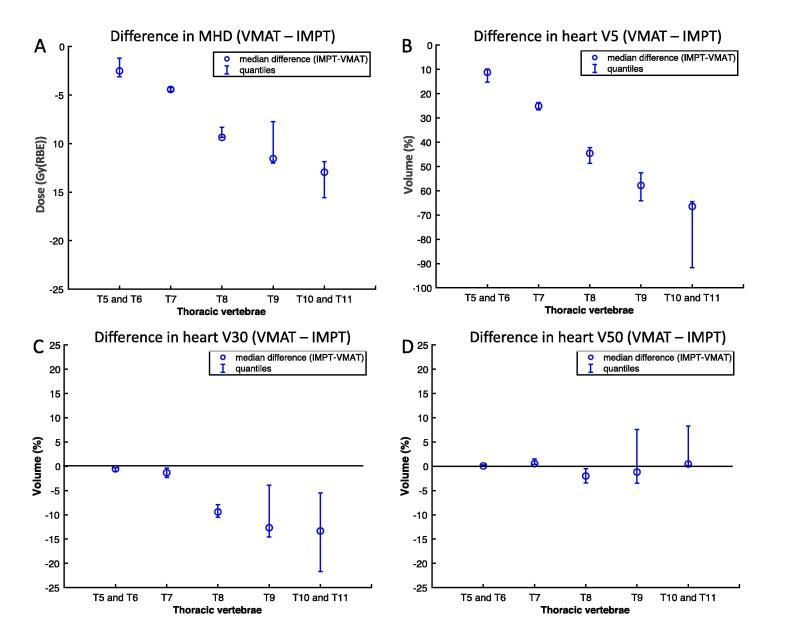
Difference in dose to the heart between VMAT and IMPT according disease extension with reference to the thoracic vertebrae.

**Fig. A2:**
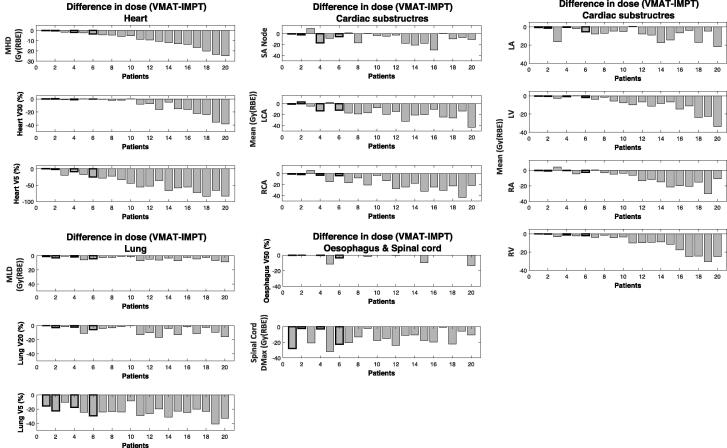
Difference in doses to OAR. Patients marked in bold box (pt 1, 2, 4 and 6) indicate those with tumour not extending to and below T7 vertebrae.

**Fig. A3:**
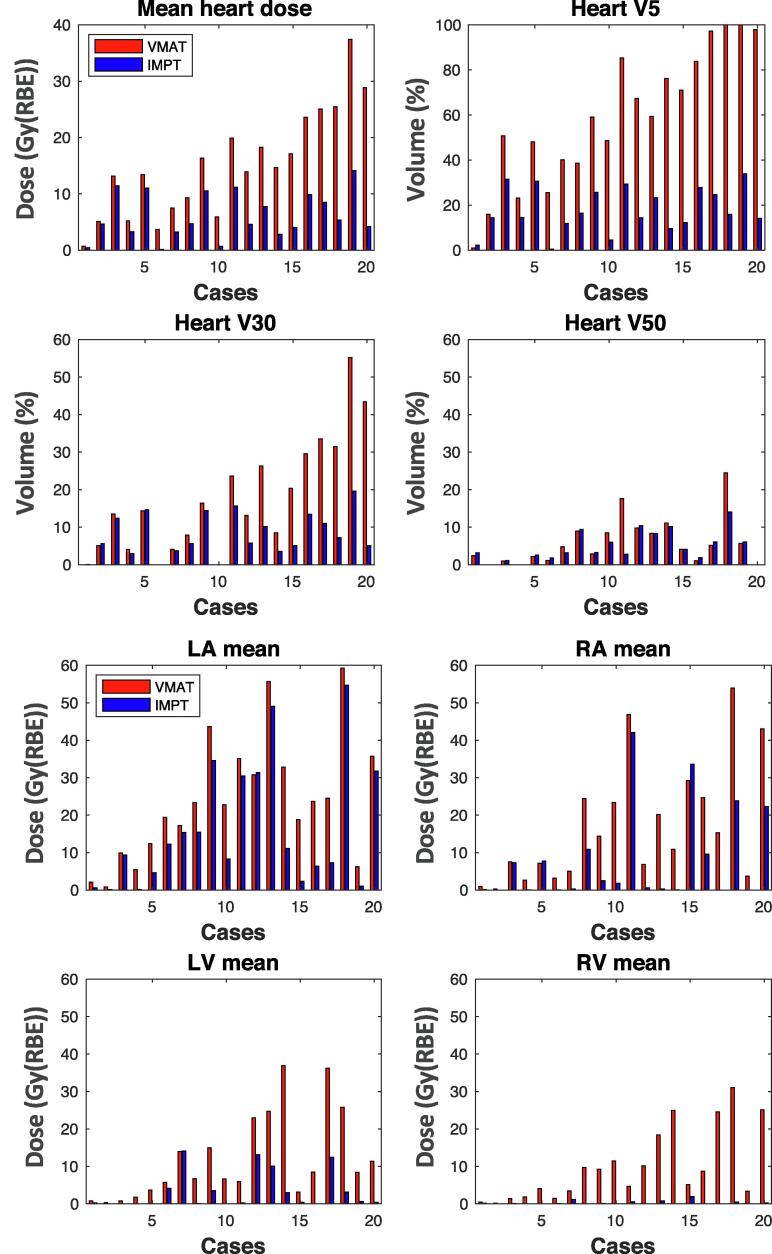
Absolute dose to the heart and substructures.

Dose to the heart and all its substuctures were significantly lower with IMPT compared to VMAT (P < .05). In VMAT plans, MHD increased as the disease extended further down the thoracic vertebral levels. Similar observations were seen for heart V5 and V30. This correlation was statistically significant in VMAT plans (MHD, V5 and V30; ρ = .67, .79, .48, P < .05), but not in IMPT plans (see Appendix Table A2). A similar trend was seen in VMAT plans for the atria (left and right, ρ = .65 and .58, P < .01) and ventricles (left and right, ρ = .68 and .64, P < .005). For structures that are immediately adjacent to the T7 thoracic vertebrae (SA node, RCA, LCA), this association was not observed (SA node, RCA, LCA, ρ = .25, .41 and .29, P = .30, .07, .22 respectively). There was a larger difference in MHD between VMAT and IMPT the lower the disease (tumour and nodal involvement) extended to with reference to the thoracic vertebrae (see Fig. 1). The absolute and difference in dose between VMAT and IMPT to the heart, its substructures and other OAR for each case can be found in Appendix Fig. A2, Fig. A3.

The median level at which the superior aspect of the heart contour started was the T7 vertebra (range: T6-T8). The difference in MHD approached 5 Gy(RBE) when the inferior part of disease overlapped the T7 vertebrae in both VMAT and IMPT plans. In this patient group, comparing between VMAT and IMPT, there was a statistically significant difference in dose (mean, V5 and V30, P < .05) to the heart and all substructures except mean dose to LCA and V30 to SA node. There was no statistically significant difference in V50 for this group of patients or the whole cohort. When the most inferior extent of disease did not overlap with the T7 vertebrae, there was no statistically significant difference in dose to the whole heart or substructures for any of the dose metrics evaluated (mean, V5, V30 and V50). A summary of the dose indices for patients with disease extension to and below T7 is found in Table 2.

**Table 2:** Median dose indices of OAR for VMAT and IMPT plans where tumour extended to or below the T7 vertebra (OAR- organs-at-risk, CI – confidence interval, RA – right atrium, LA – left atrium, RV – right ventricle, LV – left ventricle, RCA – right coronary artery, LCA – left coronary artery, SA node – sino-atrial node, NS- not statistically significant). Dose indices of plans above T7 can be found in Appendix Table A3.

OAR	Metric	VMAT (range)	IMPT (range)	P value
*To and below T7 vertebrae*				
Heart	Mean (Gy(RBE))	16.7 (5.9–37.4)	6.5 (0.7–14.1)	<.001
	V50 (%)	5 (0–24)	5 (0–14)	.691 (NS)
	V30 (%)	19 (0–100)	9 (0–20)	.001
	V5 (%)	70 (39–100)	20 (5–34)	<.001

RA	Mean (Gy(RBE))	17.7 (3.2–54.0)	2.2 (0–42.0)	.001
	V50 (%)	0 (0–57)	0 (0–46)	.374 (NS)
	V30 (%)	12 (0–100)	0 (0–62)	.009
	V5 (%)	95 (1–100)	14 (0–91)	.001

LA	Mean (Gy(RBE))	24.1 (6.2–59.3)	13.8 (1.0–54.7)	.001
	V50 (%)	9 (0–75)	5 (0–60)	.308 (NS)
	V30 (%)	29 (0–98)	17 (0–83)	.005
	V5 (%)	100 (63–100)	42 (7–99)	<.001

RV	Mean (Gy(RBE))	9.5 (1.5–31.0)	0.1 (0.0–1.94)	<.001
	V50 (%)	0 (0–5)	0 (0–0)	.109 (NS)
	V30 (%)	1 (0–52)	0 (0–0)	.003
	V5 (%)	60 (7–100)	0 (0–11)	<.001

LV	Mean (Gy(RBE))	9.9 (3.2–36.9)	1.8 (0.0–14.1)	.001
	V50 (%)	0 (0–30)	0 (0–10)	.043
	V30 (%)	3 (0–72)	1 (0–19)	.013
	V5 (%)	59 (7–100)	7 (0–42)	<.001

RCA	Mean (Gy(RBE))	21.7 (16.3–27.2)	0.1 (0.0–11.9)	.001
	V50 (%)	0 (0–11)	0 (0–0)	.317 (NS)
	V30 (%)	0 (0–100)	0 (0–0)	.028
	V5 (%)	100 (0–100)	0 (0–98)	.001

LCA	Mean (Gy(RBE))	31.5 (3.2–49.5)	13.3 (0.0–72.7)	.679 (NS)
	V50 (%)	0 (0–70)	0 (0–77)	.500 (NS)
	V30 (%)	46 (0–100)	0 (0–100)	.013
	V5 (%)	100 (43–100)	26 (0–100)	.001

SA node	Mean (Gy(RBE))	37.5 (0.2–72.6)	16.5 (0.0–72.7)	.020
	V50 (%)	9 (0–100)	0 (0–100)	.735
	V30 (%)	82 (0–100)	16 (0–100)	.091 (NS)
	V5 (%)	100 (0–100)	90 (0–100)	.007

Non-GTV lungs	Mean (Gy(RBE))	16.3 (9.8–24.9)	12.7 (8.4–17.9)	<.001
	V20 (%)	28 (16–45)	22 (15–33)	<.001
	V5 (%)	55 (32–79)	32 (22–46)	<.001

Oesophagus	V50 (%)	15 (0–55)	8 (0–56)	.875 (NS)
Spinal Cord	DMax (Gy(RBE))	42.7 (18.3–48.8)	25.9 (0.7–46.8)	<.001

### Risk of toxicity

**Fig. 2:**
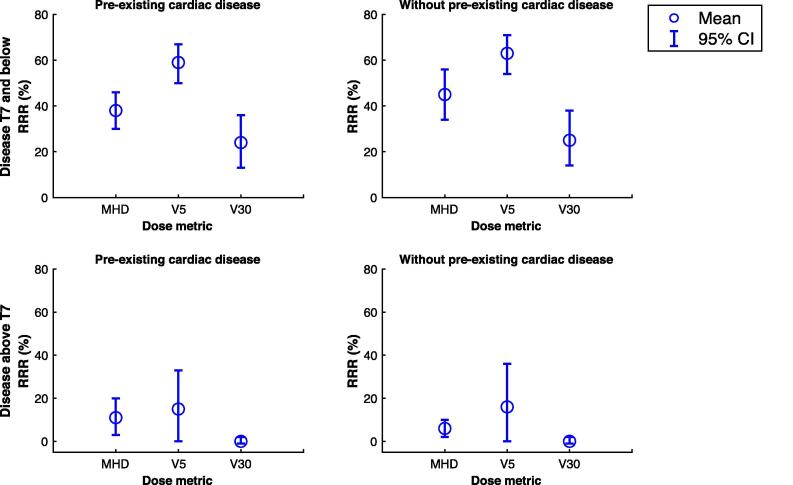
Relative risk reduction (RRR) based on presence or absence of pre-existing heart disease and dose metrics.

**Fig. A4:**
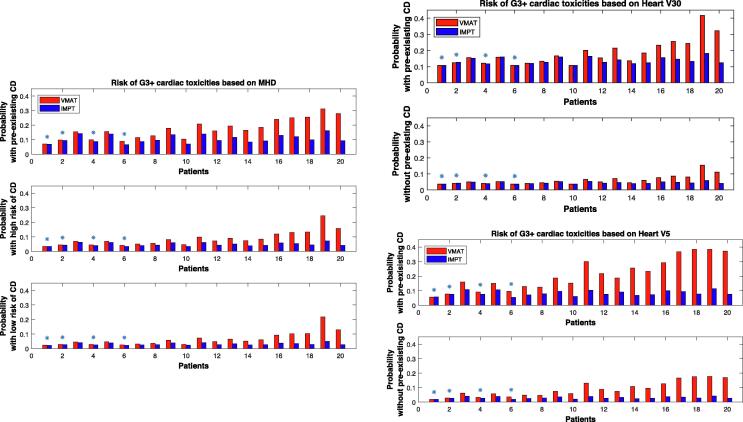
Risk of grade ⩾3 cardiac toxicities based on MHD. Asterisks indicate patients with disease not extending to and below T7 vertebrae.

The risk of cardiac complication was highest in patients with pre-existing HD and when disease overlapped with or was inferior to the T7 vertebrae. A summary of the absolute and relative risk reduction for the different scenarios is found in Table 3. For the patients in the highest risk group, the relative risk reduction (RRR) between proton and photon therapy based on MHD, V5 and V30 was 38% (95%CI 30–46%), 59% (50–67%) and 24% (13–36%), see Fig. 2). In the absence of pre-existing HD, similar RRR were observed. However, the absolute benefit was more than twofold lower for IMPT. There was limited RRR if the tumour did not extend below T7 vertebrae (RRR range:0–16%). An estimate of risk for each case can be found in Appendix Fig. A4.

**Table 3:** Risk estimates of grade⩾3 cardiac toxicities. High risk of heart disease defined as Framingham score of ⩾20% (CI – confidence interval, HD – heart disease, AR – absolute risk, MHD – mean heart dose, RRR – relative risk reduction).

	AR (%, 95% CI)		RRR (%, 95% CI)
Metric	VMAT	IMPT	
**To and below T7 vertebrae**			
*Pre-existing HD*			
MHD	19 (16–22)	11 (10–12)	38 (30–46)
Heart V5	24 (20–29)	9 (8–10)	59 (50–67)
Heart V30	23 (17–32)	14 (13–15)	24 (13–36)
			
*No pre-existing HD*			
MHD	7 (5–10)	3 (3–4)	45 (34–56)
Heart V5	10 (8–13)	3 (3–4)	63 (54–71)
Heart V30	9 (5–14)	5 (4–5)	25 (14–38)
			
*High risk of HD*			
MHD	10 (8–12)	5 (5–6)	41 (31–50)

**Above T7 vertebrae**			
*Pre-existing HD*			
MHD	9 (8–10)	8 (7–9)	11 (3–20)
Heart V5	8 (6–9)	7 (6–8)	15 (0–33)
Heart V30	12 (11–12)	11 (11–12)	0 (−1–2)
			
*No pre-existing HD*			
MHD.	3 (2–3)	2 (2–3)	6 (2–10)
Heart V5	3 (2–3)	2 (2–3)	16 (0–36)
Heart V30	4 (4–4)	4 (4–4)	0 (−1–2)
			
*High risk of HD*			
MHD	4 (4–5)	4 (3–4)	8 (2–15)

## Discussion

We have shown that IMPT can reduce heart dose compared to VMAT. The estimated clinical benefit is higher in patients with pre-existing HD and where the disease overlapped with or extended to the most superior aspect of the heart contour. The median level of the superior aspect of the heart contour began at the level of the T7 vertebra. In this patient group, the RRR of grade⩾3 cardiac toxicity was between 24 and 60%. Depending on the dose metric used, the estimated risk of complications differs. The RRR was highest using heart V5 and lowest using V30.

Radiotherapy is known to increase the long-term risk of HD. This association is well-established in breast cancer [26] and lymphoma [27]. Following the publication of the results of RTOG-0617 trial, the link between radiotherapy for lung cancer and cardiac toxicity has been increasingly recognised. However, the pathophysiology of RIHD in this context is not well understood. The risk of cardiac toxicity is unlikely to be dependent on a single dose-volume parameter. It would appear that both high dose to a small volume of heart and low dose to a large volume are likely to be important [28], [29]. Dose to the whole heart [2], [3] and sub-structures [7], [30] have been linked to survival. Current evidence point to the base-of-the-heart and left ventricle as being the most dose-sensitive regions.

PBT has the potential to reduce toxicity to the heart through reduction in heart dose. Despite the low power we were able to demonstrate statistical significance. This was due to the large differences between the groups. As the statistical test suggested that the findings were not just due to chance, we are confident that this represents a genuine effect. However, access to this technology is limited and therefore patient selection is crucial to maximise benefit of PBT. Trials of equivalent doses in unselected patient groups are unlikely to show an advantage for protons. In fact, one would anticipate similar local control and toxicity rates. For instance, when comparing oesophageal dose (see Table 2), both IMPT and VMAT would be expected to result in similar rates of oesophagitis. The benefit of PBT is likely to be related to reduction in integral dose and therefore patient selection where this advantage can be drawn on is critical. Although, our analysis showed that IMPT could potentially reduce cardiac toxicity due to lowering of heart exposure to the medium-to-low dose range, there was little reduction in the high-doses volume to the heart. Therefore, PBT may not reduce toxicity when it is associated with high dose to the heart or its substructures.

There are a number of limitations to our study. Firstly, the NTCP model that was used was derived from retrospective data from a single institution. The true incidence of cardiac toxicity following radiotherapy for lung cancer is currently unknown. It is possible that not all cardiac complications were captured. Current published data is likely to be an underestimation, especially for grade 5 toxicity, as accurate documentation of cause of death is challenging in these patients [31]. Secondly, the model was derived from a cohort of patients treated with 3D-conformal radiotherapy. Furthermore, the model by Dess et al. has not been validated and we recognize that this is a limitation of the model. However, it gives a plausible explanation for the observed decreased in overall survival in RTOG-0617 and multiple studies have since reported the association between cardiac toxicity and lung radiotherapy [28], [29]. Unfortunately, as highlighted in a recent review by Zhang et al., there are weaknesses in the literature [29]. These studies are heterogeneous in nature with inconsistencies in terms of the specific dose parameter tested. The merit of our planning study is that we have identified a subgroup of patients where specific dose volume parameters for the heart and its substructures are significantly lower in IMPT compared to VMAT. It is known that cardiovascular disease impacts on survival of lung cancer patients [32], [33]. Therefore, to our knowledge, this is the best complication model to date which incorporates baseline cardiac risk as well as dose metrics.

Another limitation is that, the model lacks consideration of lung dose metric. A number of reports have emerged suggesting the possible synergistic effect between heart and lung toxicity following lung cancer radiotherapy [34], [35]. A preclinical study has shown the likely mechanism of action being mutual cardiopulmonary dysfunction following combined cardiac and lung irradiation compared to irradiation of the heart or lung alone [34], current clinical reports are conflicting [36], [37], [35]. Finally, with the new standard of care of the addition of an immune checkpoint inhibitor following chemoradiotherapy, an updated model is needed [38].

We assumed an averaged proton RBE value of 1.1 relative to photons based on RBE values measured in vivo. We recognize that microscopically this concept breaks down and that, RBE significantly increases towards the distal end of a spread out Bragg peak [39]. Unfortunately, considerable uncertainties exist in translating in vitro and in vivo data to a clinical RBE. Therefore, given the paucity of published clinical data indicating that the average RBE of 1.1 is incorrect and lack of validated RBE models for proton therapy planning [40], [41], for the purpose of the study, we have assumed an averaged relative proton of RBE of 1.1 to photon therapy.

We recognise that the relevance of photon NTCP models to proton therapy has not been established. However, our analysis is useful in giving some indication of the likely clinical benefit of PBT in specific situations. Using an easily identifiable surrogate marker, the T7 vertebrae, one could propose a randomised VMAT vs IMPT trial in locally advanced lung cancer where the primary endpoint is cardiac toxicity. Enrichment of the study population could be achieved by only enrolling patients with stable pre-existing HD or at high risk of heart disease. A health economics evaluation should be embedded within such a trial given the cost of the technology.

However, there are many challenges in conducting a PBT trial in lung cancer. A number of lessons have been learnt from the published passive scatter proton therapy (PSPT) vs intensity modulated radiotherapy (IMRT) trial in lung cancer [12]. Overall there was no statistically significant difference in grade⩾3 pneumonitis rate. However, reduction in dose to the heart at all dose levels was reported. There were improvements in the primary endpoints of pneumonitis and local failure as the trial progressed, in particular for the proton arm. The trial highlights the importance of experience in treatment planning. Other treatment planning considerations include: the dose calculation engine, robust planning and evaluation, and motion management. Finally, not to be overlooked is the need for adaptive planning and strict radiotherapy quality assurance. These technical issues are critical in PBT relative to photon therapy due to the sensitivity of PBT plans to perturbations.

In conclusion, our analysis suggests that IMPT could benefit patients with locally advanced NSCLC whose primary tumour and nodal spread overlapped with or is inferior to T7 vertebrae compared to VMAT. The greatest benefit was seen in patients with pre-existing heart disease followed by those at high-risk of heart disease. In the highest risk group, the RRR of grade⩾3 cardiac complications was between 40 and 60%.

## Conflict of interest

The author has no conflicts of interest.

## Figures and Tables

**Table A1:** Summary of beam arrangements and range shifter use. (deg – degree, RS – range shifter in water equivalent thickness).

Plan	Gantry angle (deg)	RS (cm)
1	30	none
	110	none
	155	none
2	0	4.0
	90	4.0
	180	4.0
3	40	7.5
	220	7.5
	300	7.5
4	0	4.0
	210	4.0
	270	4.0
5	45	none
	100	none
	315	none
6	190	none
	235	none
	280	none
7	225	4.0
	270	4.0
	305	4.0
8	45	4.0
	90	none
	135	none
9	180	4.0
	200	4.0
	245	none
10	45	7.5
	120	7.5
	180	7.5
11	90	4.0
	135	4.0
	180	4.0
12	180	none
	215	none
	250	none
13	90	4.0
	135	4.0
	180	4.0
14	180	4.0
	215	4.0
	260	none
15	180	none
	180	4.0
	220	none
	270	none
16	90	4.0
	140	4.0
	180	4.0
17	180	4.0
	225	4.0
	270	4.0
18	90	4.0
	135	4.0
	180	4.0
19	270	4.0
	230	4.0
20	90	none
	135	none
	180	none

**Table A2:** Summary of Spearman correlation between heart dose and thoracic vertebrae level in VMAT and IMPT.

		VMAT		IMPT	
OAR	Metric	Spearman’s ρ	P	Spearman’s ρ	P
Heart	MHD	.67	.001	.40	.08
	V30	.48	.032	.40	.084
	V5	.79	<.001	.35	.131
RA	Mean	.65	.002	.25	.297
LA	Mean	.58	.007	.35	.135
RV	Mean	.68	.001	.17	.484
LV	Mean	.64	.002	.36	.115
RCA	Mean	.41	.07	-.04	.856
LCA	Mean	.29	.221	-.12	.620
SA node	Mean	.25	.298	.16	.504

**Table A3:** Median dose indices of OAR for VMAT and IMPT plans above T7 vertebrae (OAR – organs-at-risk, CI – confidence interval, RA – right atrium, LA – left atrium, RV – right ventricle, LV – left ventricle, RCA – right coronary artery, LCA – left coronary artery, SA node – sino-atrial node, NS – not statistically significant).

OAR	Metric	VMAT (range)	IMPT (range)	P value
*Above T7 vertebrae*				
Heart	Mean (Gy(RBE))	4.4 (0.7–5.2)	1.9 (0.1–4.6)	.068
	V50 (%)	0 (0–2)	1 (0–3)	.180
	V30 (%)	2 (0–5)	2 (0–6)	1.000
	V5 (%)	20 (0–26)	8 (0–14)	.144

RA	Mean (Gy(RBE))	1.8 (0.3–7.5)	0.1 (0–7.2)	.068
	V50 (%)	0 (0–1)	0 (0–0)	.317
	V30 (%)	0 (0–9)	0 (0–3)	1.000
	V5 (%)	5 (0–27)	0 (0–39)	.317

LA	Mean (Gy(RBE))	3.8 (0.8–9.9)	0.4 (0.1–9.4)	.068
	V50 (%)	2 (0–7)	4 (0–9)	.109
	V30 (%)	1.9 (0–8)	0 (0–0)	.317
	V5 (%)	18 (0–51)	0 (0–36)	.285

RV	Mean (Gy(RBE))	1.0 (0.2–1.9)	0.1 (0–0.2)	.068
	V50 (%)	0 (0–0)	0 (0–0)	1.000
	V30 (%)	0 (0–0)	0 (0–0)	1.000
	V5 (%)	3 (0–13)	0 (0–0)	.180

LV	Mean (Gy(RBE))	0.8 (0.3–1.8)	0.0 (0–0.2)	.068
	V50 (%)	2 (0–11)	0 (0–1)	.655
	V30 (%)	0 (0–0)	0.0 (0–0)	1.000
	V5 (%)	0 (0–11)	0 (0–0)	.317

RCA	Mean (Gy(RBE))	2.7 (0.6–3.6)	0.2 (0–0.7)	.068
	V50 (%)	0 (0–0)	0 (0–0)	1.000
	V30 (%)	0 (0–0)	0 (0–0)	1.000
	V5 (%)	9 (0–21)	0.0 (0–0)	.180

LCA	Mean (Gy(RBE))	10.3 (2.3–15.0)	0.2 (0.0–28.3)	.715
	V50 (%)	0 (0–0)	0 (0–0)	1.000
	V30 (%)	0 (0–0)	0 (0–6)	.655
	V5 (%)	84 (0–100)	6 (0–71)	.285

SA node	Mean (Gy(RBE))	3.4 (0.7–46.7)	0.2 (0.0–25.8)	.715
	V50 (%)	0 (0–29)	0 (0–0)	.317
	V30 (%)	0 (0–99)	0 (0–26)	.317
	V5 (%)	19 (0–100)	0 (0–100)	0.317

Non-GTV lungs	Mean (Gy(RBE))	11.5 (6.9–14.7)	9.0 (5.7–10.3)	.068
	V20 (%)	19 (10–24)	15 (11–20)	.144
	V5 (%)	45 (34–55)	23 (18–29)	.068

Oesophagus	V50 (%)	15 (3–35)	14 (0–34)	.465
Spinal Cord	DMax (Gy(RBE))	42.8 (30.0–47.0)	30 (1.8–44.6)	.068

## Appendices

### Treatment planning- accounting for setup and range uncertainties

In photon plans, setup errors are accounted for by adding a margin to the treatment volume to produce a PTV [42]. In proton planning, it is necessary to account for range uncertainty as well as setup uncertainty. Unfortunately, the addition of a geometric margin in proton plans is inadequate as it fails to take into account changes in density along the beam path, upon which protons are highly dependent, resulting in a distorted dose distribution. This situation is particularly relevant to IMPT as non-uniform fields are used to produce the desired dose distribution.

Despite this, many comparative studies between proton and photons have used the same CTV and PTV margin for both modalities. In passive scatter proton therapy, use of beam-specific distal and proximal margin as suggested by Moyers et al. [43] is routine [12]. A similar approach can be applied to single-field optimisation IMPT [44], [45]. However, the same concept cannot be applied in multi-field optimisation IMPT due to the non-uniform fields produced. Margins could improve target coverage at the edges of the target volume but not within the target itself. Unfortunately, they have little effect on the robustness of a plan where steep dose gradients exist within the clinical target volume (CTV) [46]. Recently, robust optimisation techniques have been developed to take into account setup and range uncertainty within the IMPT optimisation algorithm. Using this method, instead of the TPS optimising on the PTV to generate a treatment plan, the TPS optimises based on the CTV with the planner providing setup and range uncertainty parameters. In the Raystation TPS, a mini-max robust optimisation method is used whereby the TPS optimises based on the worst case scenario that could occur. This has been shown to provide robust coverage of the target compared to the conventional method of adding margins [47]. Furthermore, a comparison study between conventionally-optimised VMAT versus robustly optimised IMPT plans by Inoue et al. [10] have shown that robustly optimised plans for locally advanced NSCLC are only minimally affected by setup and range uncertainties, breathing motion, and interplay effects [10].

A 5 mm PTV expansion for VMAT plans was chosen based on our institution’s planning protocol in locally advanced lung cancer. The CTV-PTV margins were calculated using 2.5Σ+0.7α, where Σ is the population setup mean systematic error and α is the corresponding population mean random error [48]. Our institution’s mean population systematic errors, Σ, were 1.0 mm, 1.3 mm, and 0.8 mm and random errors, α, were 2.3 mm, 2.7 mm, and 2.3 mm in the x-, y- and z- directions respectively. This was calculated from recorded daily on-line shifts of patients who underwent radical radiotherapy for lung cancer at our institution. The maximum calculated distance defined the CTV-PTV isotropic margin (5 mm).

Within Raystation TPS, setup errors are specified in terms of the maximum shifts of the isocentre position [49]. Therefore, we have chosen 3 mm for the robust optimisation parameters for setup error in IMPT plans based on the threshold for online shifts at our institution.

Following plan optimisation and final dose calculations, plan robustness against setup uncertainty was performed using a probabilistic method [50]. Fredriksson et al. [47] assessed plan robustness against range uncertainty using an uncertainty parameter of 3% and compared different planning techniques in different tumour sites including lung and found the mini-max robust optimisation method to provide robust target coverage.
